# Induction of hepatocyte‐like cells from human umbilical cord‐derived mesenchymal stem cells by defined microRNAs

**DOI:** 10.1111/jcmm.13027

**Published:** 2016-11-22

**Authors:** Xia Zhou, Lina Cui, Xinmin Zhou, Qiong Yang, Lu Wang, Guanya Guo, Yu Hou, Weile Cai, Zheyi Han, Yongquan Shi, Ying Han

**Affiliations:** ^1^ State Key Laboratory of Cancer BiologyXijing Hospital of Digestive DiseasesThe Fourth Military Medical UniversityXi'anShaanxi ProvinceChina

**Keywords:** mesenchymal stem cells, miRNA, hepatic differentiation, CCl_4_, BALSS

## Abstract

Generating functional hepatocyte‐like cells (HLCs) from mesenchymal stem cells (MSCs) is of great urgency for bio‐artificial liver support system (BALSS). Previously, we obtained HLCs from human umbilical cord‐derived MSCs by overexpressing seven microRNAs (HLC‐7) and characterized their liver functions *in vitro* and *in vivo*. Here, we aimed to screen out the optimal miRNA candidates for hepatic differentiation. We sequentially removed individual miRNAs from the pool and examined the effect of transfection with remainder using RT‐PCR, periodic acid—Schiff (PAS) staining and low‐density lipoprotein (LDL) uptake assays and by assessing their function in liver injury models. Surprisingly, miR‐30a and miR‐1290 were dispensable for hepatic differentiation. The remaining five miRNAs (miR‐122, miR‐148a, miR‐424, miR‐542‐5p and miR‐1246) are essential for this process, because omitting any one from the five‐miRNA combination prevented hepatic trans‐differentiation. We found that HLCs trans‐differentiated from five microRNAs (HLC‐5) expressed high level of hepatic markers and functioned similar to hepatocytes. Intravenous transplantation of HLC‐5 into nude mice with CCl_4_‐induced fulminant liver failure and acute liver injury not only improved serum parameters and their liver histology, but also improved survival rate of mice in severe hepatic failure. These data indicated that HLC‐5 functioned similar to HLC‐7 *in vitro* and *in vivo*, which have been shown to resemble hepatocytes. Instead of using seven‐miRNA combination, a simplified five‐miRNA combination can be used to obtain functional HLCs in only 7 days. Our study demonstrated an optimized and efficient method for generating functional MSC‐derived HLCs that may serve as an attractive cell alternative for BALSS.

## Introduction

Liver transplantation is considered the only definitive treatment for liver failure, particularly, the treatment of acute liver failure (ALF), which has a high mortality. However, widespread transplantation has been restricted by the shortage of donor organs, the great cost of the process and the adverse complications related to surgery 1. A BALSS employing functional hepatocytes is a promising bridge to transplantation 2, 3. Patients with ALF can benefit from temporary liver support system and can even regenerate healthy liver tissue. Currently, primary human hepatocytes are the ideal cells for a BALSS. However, primary human hepatocytes are also in short supply and cannot be expanded *in vitro* 4. Thus, due to the limitations of this cell source, the current techniques for their cultivation and preservation, the severe immunological rejection by host immune system 5, it is urgent to discover an alternative and adequate supply of suitable hepatocytes.

MSCs possess plasticity and multidirectional differentiation potential, as first reported by Friedenstein 6. These cells can be obtained from many mesenchymal and connective tissues, such as the bone marrow, adipose tissue, peripheral blood and even the umbilical cord (UC) 7. MSCs derived from human umbilical cords (hMSCs) have attracted much attention due to their proliferative ability, easy accessibility, lack of ethical difficulties and low level of immunogenicity 8, 9. Hepatocyte‐like cells (HLCs) generated from MSCs would be desirable alterative cells for a BALSS. Most *in vivo* studies of HLCs have obtained inspiring results showing that these cells not only improved serum parameters, but also recovered the liver function *in vivo* 10, 11. To date, four main protocols have been used to induce the trans‐differentiation of MSCs into HLCs, as follows: the addition of cytokines and growth factors 10, 12, genetic modification 13, adjustment of the micro‐environment and changes in physical parameters 14, 15. The traditional method of inducing the differentiation of HLCs which relies heavily on the application of growth factors and requires long induction periods 12, does not meet the requirements for a BALSS which calls for large numbers of functional cells in a short time. Moreover, the high cost of growth factors restricted the use of this protocol 16. The purpose of our study was to explore an optimized and efficient method of inducing MSCs to differentiate into functional HLCs.

MicroRNAs are key players in cell differentiation and proliferation at the post‐transcriptional level. For example, microRNA‐125b regulates the osteogenic differentiation of MSCs *in vitro* by targeting Cbfβ 17. MicroRNA‐26a was reported to promote myoblast differentiation during skeletal muscle development and regeneration after injury by targeting the transforming growth factor b/bone morphogenetic protein (TGF‐b/BMP) signalling pathway 18. Moreover, microRNAs have recently been found to be critical regulators during the development of liver 19, 20. MicroRNA‐122, the dominant hepatocyte‐specific miRNA, participates in the process of lipid metabolism 21, hepatic circadian regulation 22 and hepatitis c virus (HCV) replication 23. A study of the changes in miRNA expression that occur during mouse liver regeneration indicated the important regulatory roles of miR‐21 and miR‐378 24.

In a previous study, we compared the miRNA expression levels of human umbilical cord‐derived MSCs (hMSCs) and hepatocyte growth factor (HGF)‐induced hepatocytes using chip analysis. We identified six miRNAs (miR‐1246, miR‐1290, miR‐148a, miR‐30a, miR‐424 and miR‐542‐5p) that were overexpressed, which were also confirmed by quantitative reverse‐transcription polymerase chain reaction during the hepatic differentiation 25. We combined the liver‐enriched microRNA miR‐122 and the six specific microRNAs with overexpression profiles during hepatic differentiation and transfected them into hMSCs, which proved to be a new method for obtaining functional hepatocytes for liver disease treatment. We not only clarified the instructive roles of these microRNAs during hepatic differentiation, but also demonstrated that the induced HLCs played exciting *in vivo* role in CCl_4_‐induced liver injury mouse model 26. Despite the promising results of our studies, we are not certain whether each miRNA in the seven‐miRNA combination is essential for the hepatic differentiation of hMSCs and do not know what the regulation mechanisms underlie this process. To explore these issues, the current study was designed to determine which microRNAs are critical to inducing the hepatic differentiation of hMSCs and ensure that the resultant cells promoted the improvement of the liver injury animal model.

## Materials and methods

### Cell culture and flow cytometric analysis

Human umbilical cord‐derived MSC were isolated according to the previously described protocol. After receiving the appropriate written consent, MSC harvested from full‐term delivery UCs. The isolated MSCs were cultured with Mesenchymal Expansion Medium (R&D Systems Inc., Minneapolis, MN, USA) in a 5% CO_2_ incubator at 37°C. For flow cytometric analysis, the cells were incubated with the following antibodies: anti‐human CD105‐PE (eBioscience Inc., CA, USA), anti‐human CD34‐FITC (eBioscience Inc. CA, USA), anti‐human CD31‐FITC (BD Pharmingen Inc., San Diego, CA, USA). Then, the cells were washed with PBS and were analysed with a Calibur flow cytometer (BD Pharmingen Inc.).

### Adipocyte differentiation

MSCs were plated at 2 × 10^4^ cells/cm^2^ in six‐well tissue culture plates. Adipocyte differentiation was induced when the cells reached 100% confluency or after confluency following the instructions of the kit (Cyagen Bioscience Inc., Guangzhou, China). After the cells had differentiated, they were fixed, washed with PBS and stained with 1 ml oil red O solution for 30 min. At the end, the stained cells were visualized under light microscope and images were captured.

### Osteogenic differentiation

MSCs were plated in Mesenchymal Expansion Medium at 3 × 10^3^ cells/ml in six‐well tissue culture plates which had been pre‐coated with 0.1% fibronectin (FN) (Sigma‐Aldrich Inc., St. Louis, MO, USA). Twenty‐four hours later, the medium was changed into osteogenic differentiation medium (Cyagen Bioscience Inc.). Within 2–3 weeks of incubation under this condition, the cells were fixed and later were stained with alizarin red working solution for 5 min. Finally, the stained cells were visualized and the images were acquired using light microscopy.

### Transfection of hMSCs with miRNA mimics

Mimics of miR‐122, miR‐148a, miR‐424, miR‐542‐5p, miR‐1246, miR‐1290 and miR‐30a were synthesized from RiboBio Co., Ltd. (Guangzhou, China). A random non‐targeting sequence miRNA mimic (mimic‐nc) from RiboBio Co., Ltd. was used as a negative control. For the transfections, we removed one miRNA at a time from the seven‐miRNA set and transfected the rest of them as a new set into the MSCs in each group. Based on this protocol, we divided the miRNA combination into N groups that contained (N‐1) miRNAs. One day before transfection, the MSCs were seeded in 24‐well plate at a density of 3 × 10^3^ cells/400 μl. Each mimic sample of 200 pmol miRNA mimic (10 μl of a 20 μM solution) was diluted in 50 μl of Opti‐MEM serum‐free medium (Invitrogen Inc., Carlsbad, CA, USA). Then, 1 μl of lipofectamine 2000 was incubated in 50 μl Opti‐MEM for 15 min. Subsequently, the two solutions were combined and the mixture was added to each well containing MSCs. After 8–10 hrs, the medium was replaced with Dulbecco's modified Eagle's medium (DMEM) containing serum and penicillin/streptomycin. At 7 days after transfection, the transfected cells were harvested for mRNA and protein analysis at time point.

### RNA isolation and RT‐PCR

The total RNA of the cells was isolated with the RNA Box kit (TaKaRa Biotechnology Co., Ltd., Dalian, China) according to the manufacturer's instructions. A 500‐ng aliquot of total RNA was used for cDNA synthesis with the PrimeScript RT Reagent Kit Perfect Real Time kit (TaKaRa Biotechnology Co., Ltd.). PCR amplification was performed with the SYBR Premix Ex Taq II reagent (TaKaRa Biotechnology Co., Ltd.) according to a previously published protocol and a light cycler real‐time PCR system (Roche Diagnostics Mannheim, Germany). GAPDH expression was analysed to normalize the level of target gene expression in each sample. Human U6 expression was used to normalize the level of target miRNA expression. The primers used for qRT‐PCR are shown in Table 1. The Uni‐miR qPCR primer was used as the reverse primer (TaKaRa Biotechnology Co., Ltd.) for miRNA analysis. Relative changes in gene and miRNA expression were determined with the 2^−ΔΔt^ method.

**Table 1:** Primers for RT‐PCR analysis

Gene	Primer (5′ to 3′)	Annealing temperature
GAPDH	F: GCACCGTCAAGGCTGAGAAC	61°C
	R: TGGTGAAGACGCCAGTGGA	
ALB	F:ACTGCATTGCCGAAGTGGA	60°C
	R:GCAGCACGACAGAGTAATCAGGA	
HNF4A	F:AGCTGCAGATCGATGACAATGAG	61°C
	R:CATACTGGCGGTCGTTGATGTAG	
AFP	F:TGCAGCCAAAGTGAAGAGGGAAGA	64°C
	R:CATAGCGAGCAGCCCAAAGAAGAA	
TF	F: CCTCCTACCTTGATTGCATCAG	58°C
	R: TTTTGACCCATAGAACTCTGCC	
CYP3A4	F: AAGTCGCCTCGAAGATACACA	59°C
	R: AAGGAGAGAACACTGCTCGTG	
G6P	F:TAGAGCTGAGGCGGAATGGGAG	60°C
	R: GCTGGAGTCCTGTCAGGCATTGC	
CK7	F: GCTGAGGCTGAAGCCTGGTA	61°C
	R: CATCTCTGAAATCTCATTCCGGGTA	
PDX1	F: ACTCCACCTTGGGACCTGTTTAGA	62°C
	R: CGAGTAAGAATGGCTTTATGGCAGA	
EpCAM	F: GAATGGCAAAGTATGAGAAGGCTGA	61°C
	R: TCCCACGCACACACATTTGTAA	
CYP1A1	F: GGAGCTAGACACAGTGATTGGC	60°C
	R: GGTGAAGGGGACGAAGGA	
MiR‐122	TGGAGTGTGACAATGGTGTTTG	60–62°C
MiR‐148a	TCAGTGCACTACAGAACTTTGT	
MiR‐424	CTTCCCCCCAGTAATCTTCATC	
MiR‐542‐5P	TCGGGGATCATCATGTCACGAGA	
MiR‐1246	AATGGATTTTTGGAGCAGG	
MiR‐30a	TGTAAACATCCTCGACTGGAAG	
MiR‐1290	TGGATTTTTGGATCAGGGA	

### LDL uptake and PAS staining

The LDL uptake ability of the induced hepatocytes was evaluated by incubating them in 10 mg/ml acetylated LDL labelled with 1, 19‐dioctadecyl‐3,3,39, 39‐tetramethylindo‐carbocyanine perchlorate (Dil‐Ac‐LDL) (Yiyuan Biotechnologies, Guangzhou, China) for 4 hrs. Then, the cells were washed and were visualized by fluorescence microscopy. A PAS staining kit (Baso Diagnostics Inc., Zhuhai, China) was used to analyse the glycogen storage ability of the induced hepatocytes. According to the instructions, the cells were fixed and then were incubated with periodic acid and Schiff's reagent. After washing with tap water, the cells were visualized by light microscopy.

### Urea production and ICG uptake assays

The urea production ability of the HLCs was evaluated using a commercial BUN assay kit (Nanjing Jiancheng Bioengineering Institute, Jiangsu, China) according to the manufacturer's instructions. For this analysis, the HLCs were cultured in the six‐well plate for 24 hrs in the expansion medium with or without 10 mM NH_4_Cl. Then, the cells were harvested and the absorbance at 640 nm was read using an automatic microplate reader (Bio‐Rad Laboratories, Hercules, CA, USA). To analyse the ICG uptake ability of the induced cells, cardiogreen (Sigma‐Aldrich Inc.) was dissolved in sterile ddH_2_O to prepare a 50 mg/ml stock solution. Then, the solution was diluted in DMEM and added to the cells to a final concentration of 1 mg/ml. After 30‐min. incubation and a 10‐min. water wash, the cells were visualized by light microscopy.

### CYP1A1 (EROD) activity

The activity of cytochrome P450 (CYP) was measured by the P450‐Glo CYP1A1 assay kit (Promega Corporation, Madison, WI, USA). After transfection, the cells were treated with 100 μM omeprazole for 48 hrs. Then, the medium was replaced with fresh medium containing a luminogenic CYP substrate (luciferin‐CEE) and the plates were incubated at 37°C for an appropriate period. Next, 25 μl of the culture medium from each well was transferred to a 96‐well opaque white plate at room temperature, and 25 μl of luciferin detection reagent was added to initiate the luminescence reaction. The plate was incubated at room temperature for 20 min., and then the levels of luminescence were read using a CCD camera.

### Western blotting

The cells were lysed by RIPA lysis buffer (Beyotime Inc., Shanghai, China), and the proteins present were resolved in a 10% SDS‐PAGE gel. After electrophoresis and transfer of the proteins to NC membranes with semi‐dry transfer method, the membrane was blocked in 10% non‐fat dried milk dissolved in Tris Buffered Saline with Tween (TBST) for 2 hrs and was then incubated with a primary antibody at 4°C overnight. Then, the blots were washed with TBST and were incubated with a horseradish peroxidase‐conjugated secondary antibody (diluted 1:2000; Santa Cruz Biotechnology, Santa Cruz, CA, USA) at room temperature for 1 hr. Finally, the labelled blots were analysed by Femto (Pierce, Rockford, IL, USA) following the manufacturer's instructions. The primary antibodies: human anti‐ALB (Abcam, Cambridge, England), human anti‐HNF4A (Santa Cruz Biotechnology) and human anti‐CYP3A4 (Santa Cruz Biotechnology).

### Cell transplantation

Eight‐week‐old male BALB/c nude mice were purchased from the animal centre of The Fourth Military Medical University. CCl_4_ that was diluted with olive oil to form a 20% (v/v) solution was administrated intraperitoneally to the mice at the dose of 8 μl/g to produce the ALF model. And 10% CCl_4_ was administrated in the same manner to induce the acute liver injury model. One day later, the CCl_4_‐induced liver failure mice were divided into three groups. Group A was received HLC‐5 transplantation; Group B was received HLC‐7 injection; and Group C was injected with saline as the control. A total of 1 × 10^6^ cells were injected into the tail vein of the mice. In each group, six mice were administrated for survival analysis in a week after treatment. In the same way, the serum parameters and histological conditions of the liver injury groups, each of which contained 10 mice, were analysed. Then, the serum level of albumin (ALB), alanine transaminase (ALT), aspartate aminotransferase (AST) and bilirubin (BIL) was analysed with an automatic chemistry analyser AU560 (Olympus, Tokyo, Japan) in a clinical laboratory. Fresh liver sections were prepared for H&E staining and Sirius red staining. Semiquantitative analysis of the liver collagen content was conducted by evaluating 10 randomly chosen fields at 400× magnification using Image Proplus software.

### Immunofluorescence analysis

Fresh liver tissues were fixed by 4% paraformaldehyde for 30 min. and washed with PBS. After blocking with normal goat serum for 1 hr, the sections were incubated with an anti‐human ALB (Abcam) at 4°C overnight and then were incubated with secondary antibody labelled with Alexa Fluor 488 (Invitrogen Inc.) at room temperature for 1 hr. Later, the images were acquired by a laser scanning confocal microscope.

### Statistical analysis

The data were in the expression of mean values ± S.D. A one‐way analysis of variance and a *t*‐test were performed to identify the significant differences. The Kaplan–Meier method was used for survival analysis. A *P*‐value of <0.05 was considered significant. For all statistics, the data from at least three independent samples or repeated experiments were used.

## Results

### The seven‐miRNA without‐miR‐30a set converted hMSCs into functional HLC

The MSCs isolated from human UCs exhibited a typical fibroblast‐like shape at passage 4. These MSCs were positive for the expression of CD105 (99.8%), but were negative for the expression of CD31 (0.3%) and CD34 (0.4%), which are distinctive markers of endothelial cells and hematopoietic stem cells, respectively. When cultured under standard conditions, the hMSCs could differentiate into osteoblasts and adipocytes, indicating they had multipotent differentiation ability (data shown in Fig. S1).

**Figure 1:**
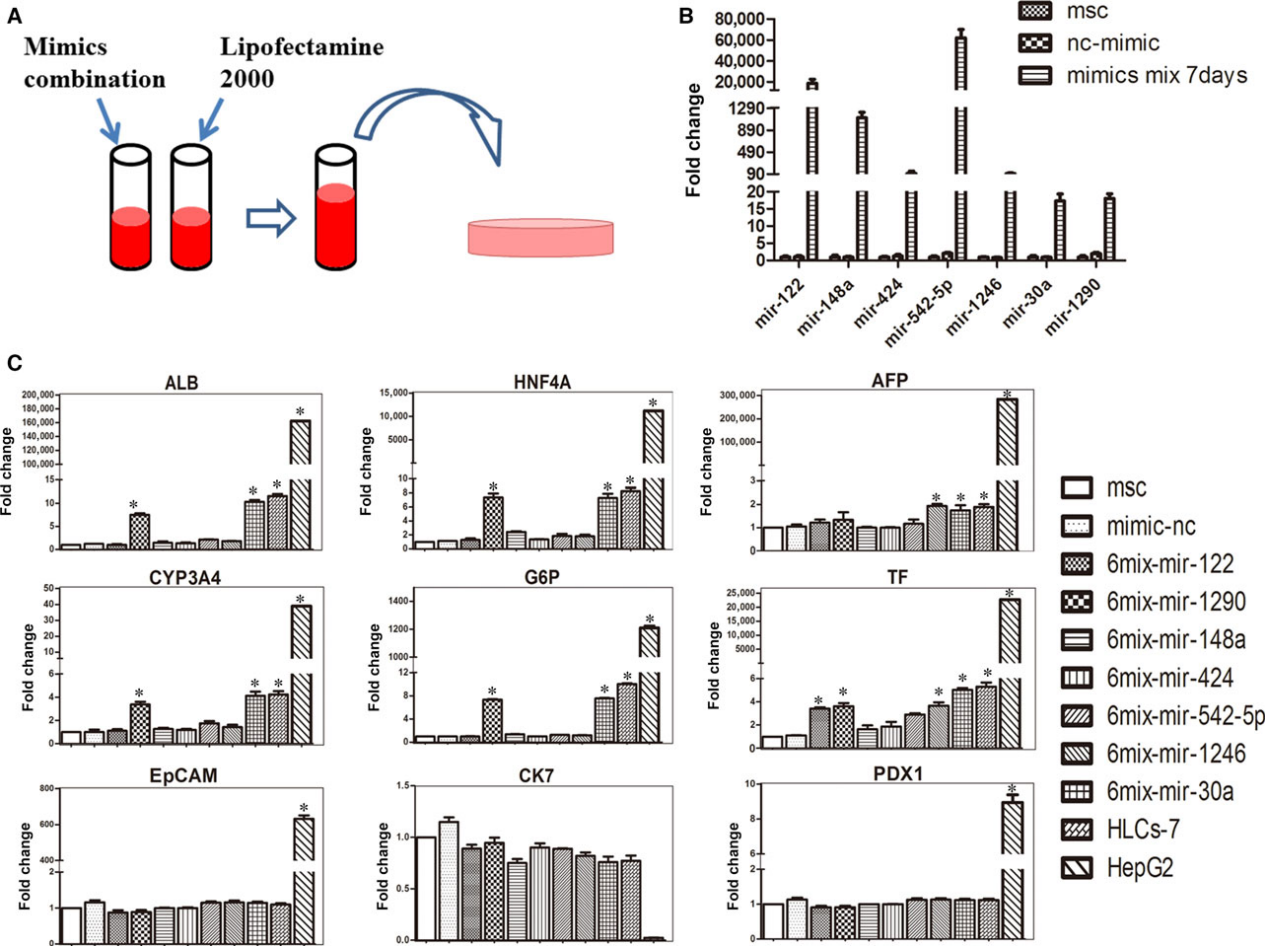
Effects of selected microRNAs on the hepatic differentiation of MSCs. (**A**) Schematic outline of the protocol for converting MSC to HLCs by transfection with miRNA mimics. (**B**) The relative expression of miRNA in hMSCs transfected with miRNA mimic combination was analysed by qPCR at day 7. (**C**) The mRNA levels of the following hepatocyte‐specific genes expression measured by qPCR at day 7: ALB, HNF4A, AFP, CYP3A4, G6P, TF, EpCAM, CK7 and PDX1. The data are presented by mean ± S.E.M. (*n* = 3), compared with mimic‐nc, *P < 0.05, ***P < 0.001 by Student's *t*‐test. HLC, hepatocyte‐like cell; ALB, albumin; AFP, a‐foetoprotein; HNF, hepatic nuclear factor; EpCAM, epithelial cell adhesion molecule; PDX1, pancreatic and duodenal homeobox 1; CK, cytokeratin; CYP, cytochrome P‐450.

In the previous study, we clarified a set of seven microRNAs (miR‐122, miR‐1246, miR‐1290, miR‐148a, miR‐30a, miR‐424 and miR‐542‐5p) successfully induced the differentiation of hMSCs into functional hepatocyte‐like cells (HLC‐7). Despite exciting results, we were uncertain whether each of these microRNAs was essential for the MSC hepatic differentiation process. In this study, to determine which of the seven microRNAs are critical for hepatic differentiation, we examined the effect of omitting individual miRNAs from the combination of seven candidate miRNAs. We synthesized the seven miRNA mimics and divided them into seven groups, each contained six different miRNA mimics (6mix) and transfected them into hMSCs (Fig. 1A). To confirm that transfection with the mimics effectively increased the relative expression levels of the corresponding miRNA in hMSCs, we tested the miRNA expression levels at 7 days after transfection. We found that the miRNA mimics miR‐122, miR‐148a, miR‐424, miR‐542‐5p, miR‐1246, miR‐30a and miR‐1290 increased relative expression of their respective miRNAs significantly, thereby resulting in the successful overexpression of these miRNAs (Fig. 1B). Surprisingly, after co‐transfection of the 6mix for 7 days, qRT‐PCR results showed that removal of 6mix set lacking miR‐30a (miR‐122, miR‐1290, miR‐1246, miR‐148a, miR‐424 and miR‐542‐5p) or miR‐1290 (miR‐122, miR‐30a, miR‐1246, miR‐148a, miR‐424 and miR‐542‐5p) induced MSCs to express liver‐specific gene ALB at a high level. However, the ALB expression level of MSCs transfected with a 6mix set lacking miR‐1290 (6mix‐without‐miR‐1290) was lower than the MSCs transfected with a 6mix set lacking miR‐30a (6mix‐without‐miR‐30a). When the other miRNAs were removed from the mixture, the ALB expression levels were not affected significantly compared with the mimics‐nc transfected group. The HLC generated from 6mix‐without‐miR‐30a set expressed other hepatic genes, including HNF4A, AFP, TF, CYP3A4 and G6P at levels that were increased by 6.2‐, 1.7‐, 5.0‐, 4.1‐ and 7.5‐fold, respectively, which were similar to the case for HLC‐7. Moreover, the induced HLC resulted from 6mix‐without‐miR‐30a set do not express the pancreatic islet marker gene PDX1, the cholangiocyte marker gene CK7 or the liver progenitor cell marker gene EpCAM, demonstrating the special role of these microRNAs in hepatic differentiation (Fig. 1C). These data indicated that with the exception of miR‐30a, the other six miRNAs played important roles in the initiation of hepatic differentiation.

**Figure 2:**
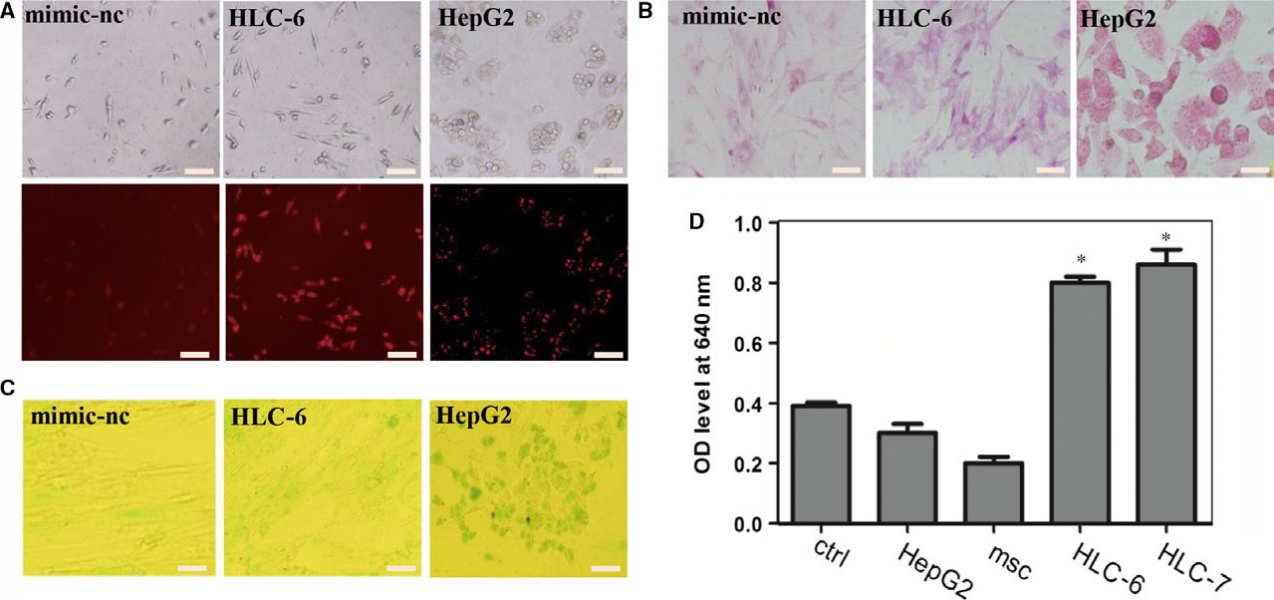
Functional analysis of the HLCs induced by a set of six miRNAs without miR‐30a (HLC‐6). (**A**) LDL uptake analysis of HLC‐6, the scale bar is 200 μm; (**B**) The ability of glycogen storage of HLC‐6 analysed by PAS staining, the scale bar is 100 μm; (**C**) ICG uptake ability of HLC‐6, the scale bar is 200 μm; (**D**) Urea production ability of HLC‐6 measured by BUN synthetic kit. The data were represented by mean ± S.D. (*n* = 3), compared with control, *P < 0.05 by Student's *t*‐test. LDL, low‐density lipoprotein; ICG, Indocyanine green; PAS, periodic acid—Schiff; BUN, blood urea nitrogen.

Next, in order to test whether the induced HLC from 6mix‐without‐miR‐30a set (HLC‐6) performed liver function *in vitro*, we conducted a series of examinations to evaluate their LDL and ICG uptake abilities, glycogen storage ability and urea production ability. Surprisingly, the results demonstrated that compared with the undifferentiated hMSCs, almost all of the HLC‐6 could take up LDL (Fig. 2A) and that the half of HLC‐6 could take up ICG (Fig. 2C). Moreover, when exposed to 10 mM ammonium chloride for 24 hrs, the HLC‐6 showed an obvious increase in their ability to produce urea (Fig. 2D). After induction for 7 days, PAS staining showed that compared with negative control group, most of HLC‐6 had stored glycogen (Fig. 2B). These results demonstrated that 6mix‐without‐miR‐30a set successfully induced hepatic differentiation of hMSCs, which demonstrated miR‐30a was dispensable for the induction of HLCs.

### The seven‐miRNA set without miR‐30a and miR‐1290 converted hMSCs into functional HLC

**Figure 3:**
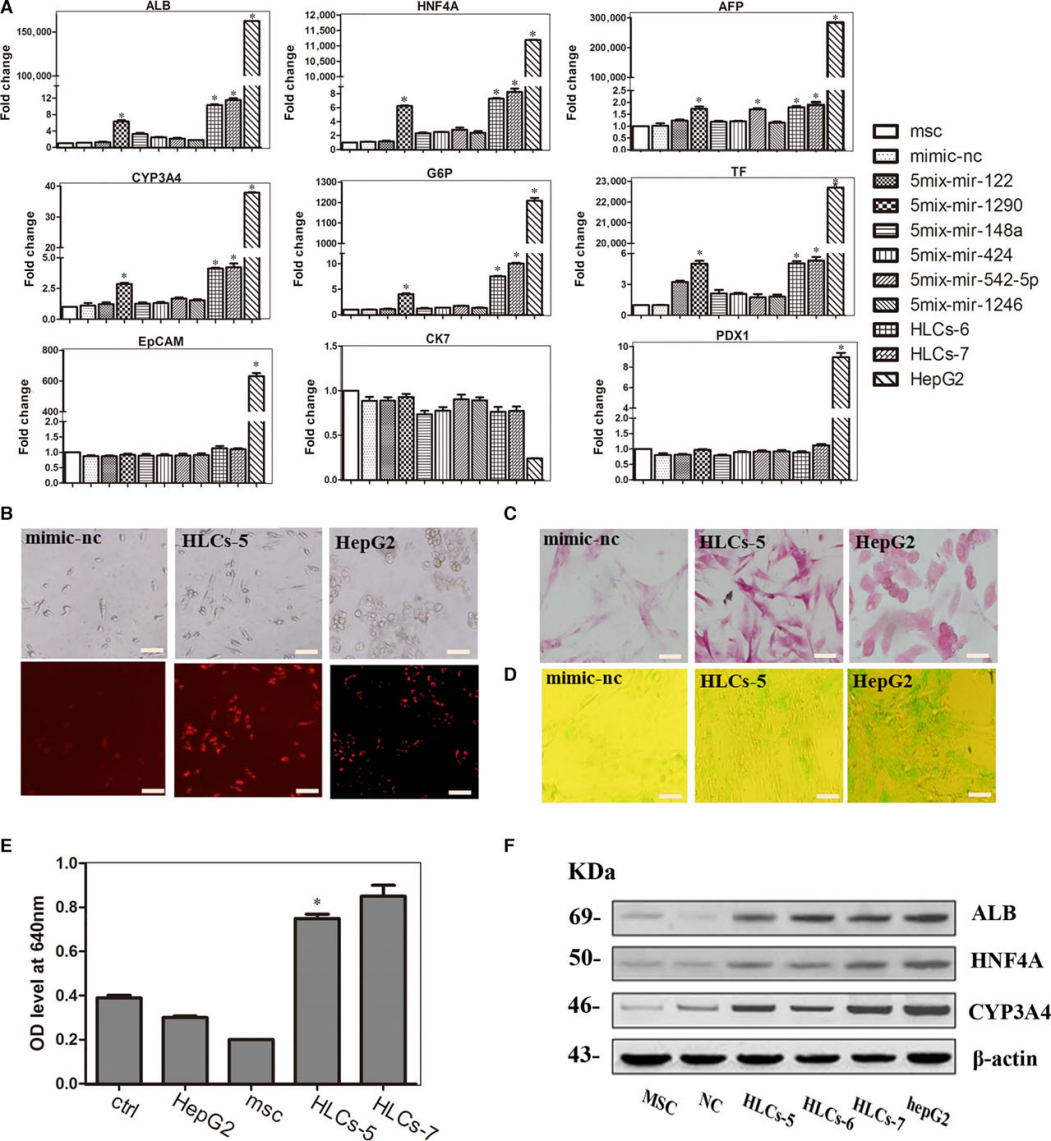
Effects of transfecting hMSCs with the five‐miRNA combination. (**A**) The expression levels of the following hepatocyte‐specific genes were determined by qPCR at day 7: ALB, HNF4A, AFP, CYP3A4, TF, G6P, PDX1, EpCAM and CK7. The data are presented by mean ± S.E.M. (*n* = 3), compared with mimic‐nc, *P < 0.05, ***P < 0.001 by Student's *t*‐test. (**B**) LDL uptake ability of HLC‐5, the scale bar is 200 μm. (**C**) PAS staining is used to measure the glycogen store ability of HLC‐5, the scale bar is 100 μm. (**D**) ICG uptake analysis of HLC‐5, the scale bar is 200 μm. (**E**) Analysis of BUN synthetic ability of HLC‐5, the data were represented by mean ± S.D. (*n* = 3), compared with control, *P < 0.05 by Student's *t*‐test. (**F**) Hepatocyte‐specific gene expression at protein level was analysed by Western blotting.

Based on the above‐described results, we wished to determine whether a combination of five microRNAs could convert hMSCs into functional hepatocytes. Thus, we removed one miRNA from the pool of 6mix‐without‐miR‐30a and overexpressed the remaining five miRNAs in each cell group. qRT‐PCR was performed to analyse the expression of liver marker genes. We were excited to find that removal of miR‐1290 from the pool of six miRNAs (leaving miR‐122, miR‐148a, miR‐424, miR‐542‐5p and miR‐1246) stimulated hMSCs to differentiate into HLCs. The HLCs induced from five miRNAs minus miR‐1290 (HLC‐5) expressed hepatocyte‐specific genes HNF4A, AFP, ALB, TF, CYP3A4 and G6P, which were increased 6.2‐, 1.7‐, 6.3‐, 5.0‐, 2.8‐ and 4.0‐fold, respectively, compared with the control level. Removing each of the other miRNA did not induce significant changes in the liver marker genes. Removing miR‐542‐5p from the miRNA pool resulted in the transfected cells with increased levels of only some of liver‐specific genes, like AFP and HNF4A. When miR‐122 was removed from the pool, only TF expression was up‐regulated, and the level was lower than 5mix‐without‐miR‐1290. Considering the overall expression of these liver markers in the transfected cells, we decided to omit miR‐1290 from the six‐miRNA combination. Besides, the HLC‐5 failed to express PDX1, CK7 and EpCAM genes (Fig. 3A). To further confirm the quality of HLC‐6 and HLC‐5 produced by microRNA combinations, we analysed the expression of ALB, HNF4A and CYP3A4 at protein level by Western blotting. The results showed that the levels of these proteins of HLC‐6 and HLC‐5 were comparable to those of HLC‐7 and hepG2, consistent with the result of qRT‐PCR analysis (Fig. 3F).

Next, we tested *in vitro* liver function of the HLC‐5 of 5mix‐without‐miR‐1290 by PAS staining, ICG and LDL uptake ability and urea production assays after transfection for 7 days. Interestingly, the results of PAS staining indicated that at 7 days after transfection, the HLC‐5 could store glycogen (Fig. 3C). Many of HLC‐5 demonstrated the ability to produce urea when exposed to ammonium chloride for 24 hrs (Fig. 3E). Moreover, approximately 40% of HLC‐5 took up ICG (Fig. 3D) and most of these cells had taken up LDL (Fig. 3B). We quantified LDL uptake, ICG uptake and PAS storage abilities of the cells. The results demonstrated that HLCs could have similar liver function with HLC‐6, and even to HLC‐7 (Fig. S2A). These results illustrated that without miR‐1290, the combination of 5mix (miR‐122, miR‐148a, miR‐424, miR‐542‐5p and miR‐1246) could induce MSCs to differentiate into HLC that exhibited liver function *in vitro*.

### No combination of four microRNAs could induce the trans‐differentiation of hMSCs into hepatocytes

**Figure 4:**
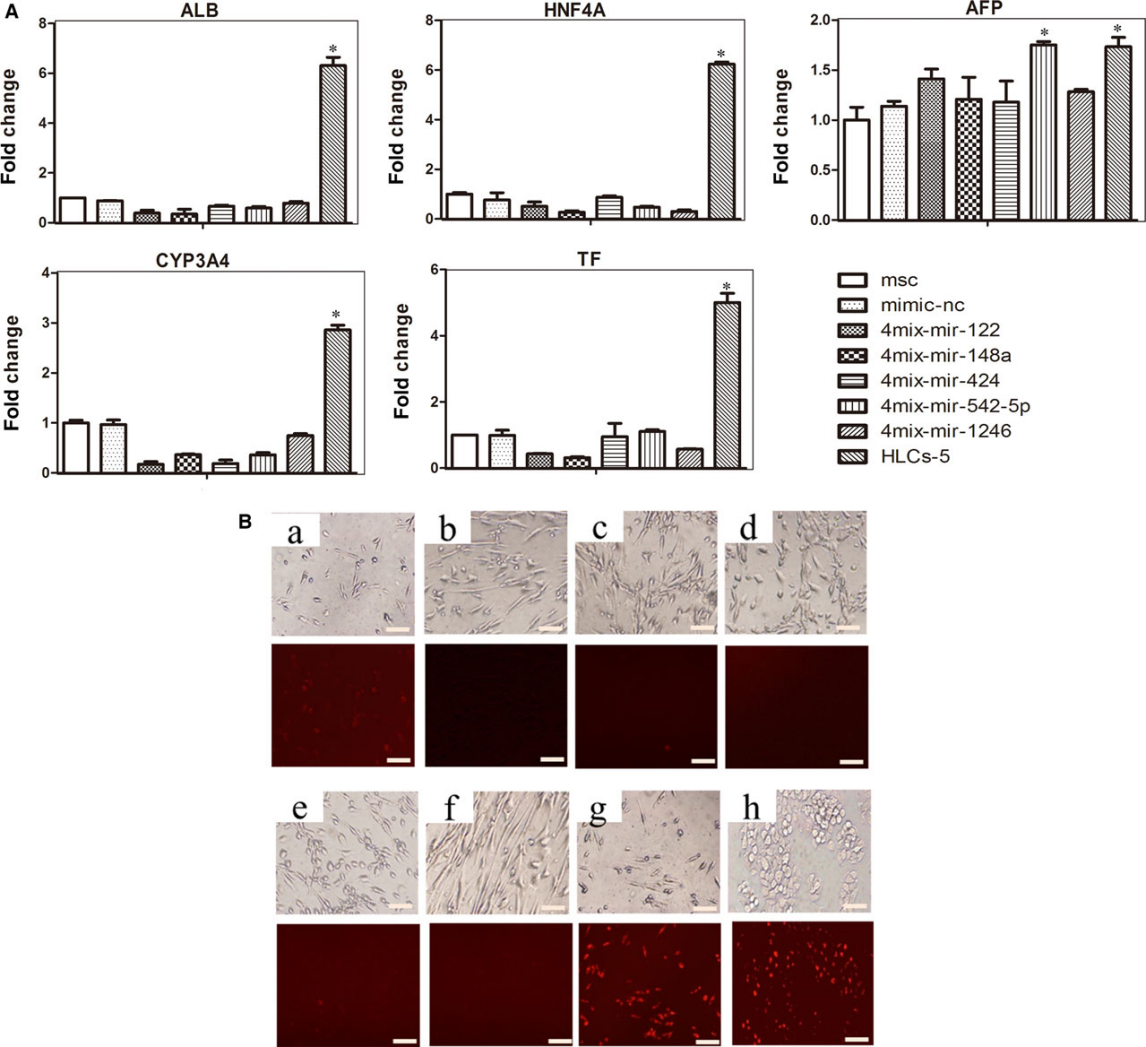
Effects of four‐miRNA combination on hMSCs. (**A**) Liver marker genes expression analysed by qPCR at day 7: ALB, AFP, HNF4A, CYP3A4 and TF. The data were represented by mean ± S.E.M (*n* = 3), compared with mimic‐nc, *P < 0.05, ***P < 0.001 by Student's *t*‐test. (**B**) Analysis of LDL uptake ability at day 7: a) mimics‐nc; b) 4mix‐without‐miR‐122; c) 4mix‐without‐miR‐148a; d) 4mix‐without‐miR‐424; e) 4mix‐without‐miR‐542‐5p; f) 4mix‐without‐miR‐1246; g) HLC‐5; h) HepG2, the scale bar is 200 μm.

We next analysed liver‐specific genes in the cells resulting from the transfection of hMSCs with all possible four‐miRNA combinations and found that no group of four‐microRNA induced hMSCs to differentiate into HLCs. After 7‐day transfection, the morphology of cells showed no obvious changes, still resembling that of the fibroblast‐like hMSCs. qRT‐PCR analysis showed that there was no significant difference in the levels of liver‐specific genes ALB and HNFA in comparison between 4mix‐transfected cells and hMSCs. Moreover, the transfected cells did not express the late liver marker CYP3A4 and TF. However, when miR‐542‐5p was omitted from the pool, the expression of AFP gene was increased, indicating AFP and miR‐542‐5p might have a regulatory relationship (Fig. 4A). We also tested the LDL uptake ability of these induced cells and observed no signs of functional HLCs, compared with those of HLC‐5 and HepG2 cells (Fig. 4B). These data indicated that removing any microRNA from the five‐miRNA pool (miR‐122, miR‐148a, miR‐424, miR‐542‐5p and miR‐1246) could not stimulate and obtain the induced hepatocytes, which meant the five‐miRNA set was the simplified combination for hepatic differentiation of MSCs.

Then, we isolated human primary hepatocytes as positive control to evaluate the features of HLC‐5. We compared the expression levels of liver marker genes in HLC‐5 and primary hepatocytes. The expression levels of these genes of HLC‐5 were lower than those of primary hepatocytes (Fig. S2C and D). Additionally, we measured the mRNA level of CYP1A1 and its (EROD) activity to evaluate the enzyme activity of HLC‐5. The qRT‐PCR result showed that HLC‐5 expressed higher level of CYP1A1 than mimic‐nc group (Fig. S2E). After omeprazole induction, the level of CYP1A1 activity was greatly increased in HLC‐5, but lower than that of primary hepatocytes (Fig. S2F). These results demonstrate that the transfection‐based method of converting MSCs into HLCs must be improved. However, it is possible that the HLC‐5 was not sufficiently mature for comparison with primary hepatocytes.

### HLCs transplantation improved the liver of CCl_4_‐induced liver failure

**Figure 5:**
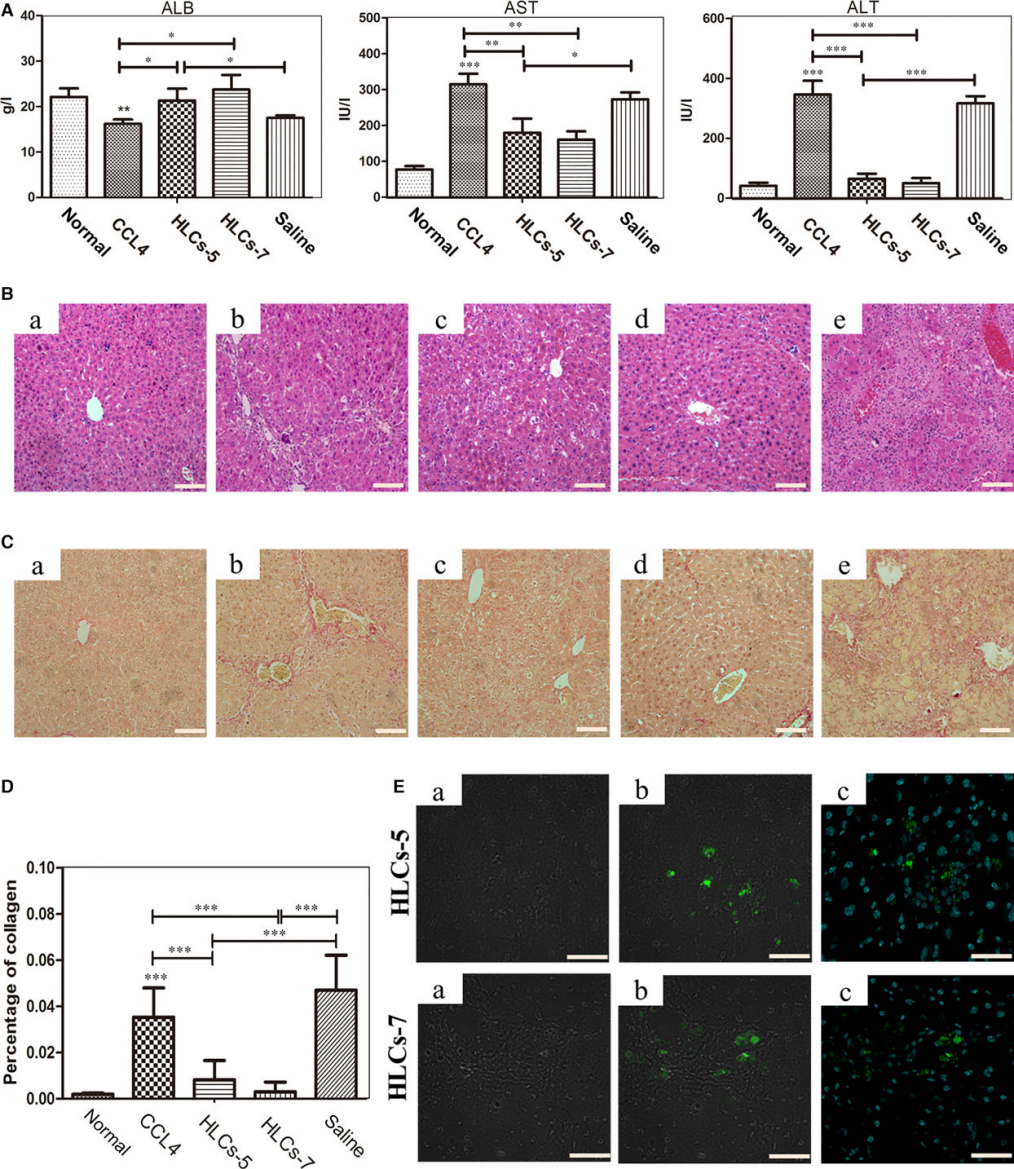
HLC‐5 transplantation improved liver function of CCl_4_‐injured nude mice (*n* = 10). (**A**) The levels of serum parameters ALB, ALT and AST of liver‐injured mice after cell transplantation were measured. (**B**) H&E staining of liver tissue from mice exposed to different treatments: a) normal; b) CCl_4_‐injured; c) HLC‐5 transplantation; d) HLC‐7 transplantation; e) saline injection. The scale bar is 50 μm. (**C**) Sirius red staining of liver tissue from mice exposed to different treatments: a) normal; b) CCl_4_‐injured; c) HLC‐5 transplantation; d) HLC‐7 transplantation; e) saline injection. The scale bar is 50 μm. (**D**) The changes in collagen content of different treatment in Sirius red staining. (**E**) Immunofluorescence staining to locate the transplanted HLC‐5 and HLC‐7: a) white; b) ALB (green); c) the merge of ALB (green); and DAPI (blue). The scale bar is 20 μm. CCl_4_, carbon tetrachloride.

To test whether human HLC‐5 derived from five‐microRNA combination had sufficient hepatic function, like that of HLC‐7, to support liver recovery from injury induced by carbon tetrachloride (CCl_4_) treatment, nude mice were injected intraperitoneally with CCl_4_ to trigger acute liver injury. We examined the values of serum parameters and performed histologic evaluations to assess the effects of the transplanted HLC‐5. Compared with normal mice, CCl_4_‐injured mice suffered from weight loss and showed significant ballooning and necrotic hepatocytes and infiltration of inflammatory cells was observed in histology. To serum parameters, the level of serum ALB decreased significantly (from 22.1 ± 1.9 g/l to 16.2 ± 0.9 g/l) in these animals, while the level of ALT and AST increased obviously. Then, we treated liver‐injured mice with HLC‐5, HLC‐7 and saline as a control. Notably, upon treatment with HLC‐5 and HLC‐7, liver‐injured nude mice showed great improvements in the values of serum parameters, especially the level of ALB (from 16.2 ± 0.9 g/l to 21.3 ± 2.6 g/l), indicating the recovery of liver function. The improvement of serum parameters in the HLC‐7 treatment group was identical to our previously reported results. In the HLC‐5 treatment group, the levels of ALB and ALT were restored to normal, and the level of AST demonstrated obvious improvement as well. Interestingly, no significant difference was observed between HLC‐5 and HLC‐7 treatment groups (Fig. 5A). We also observed reduced serum BIL levels, including the levels of total bilirubin (TBIL), direct bilirubin (DBIL) and indirect bilirubin (IDIL) in HLC treatment groups (Fig. S3A). The levels of TBIL and IBIL were affected significantly after HLCs treatment. Although there was no big difference, the level of DBIL was decreased after cell therapy (Fig. S3A). Histological analysis of H&E staining sections showed the damage of liver architecture and inflammatory cell infiltrations induced by CCl_4_ treatment. Sirius red staining demonstrated that CCl_4_ treatment greatly increased the liver collagen content. After the treatment with the induced HLC‐5 and HLC‐7, significant improvement in liver histology and a decrease in liver collagen content were observed in mice exposed to CCl_4_ (Fig. 5B–D). Then, we traced the transplanted cells in the liver section to confirm that the improvement of liver injury was resulted from the HLC transplantation. We used immunofluorescence to locate the transplanted cells in the sections of mouse livers. Human ALB‐positive transplanted cells were observed in the HLC‐5 and HLC‐7 treatment groups (Fig. 5E).

**Figure 6:**
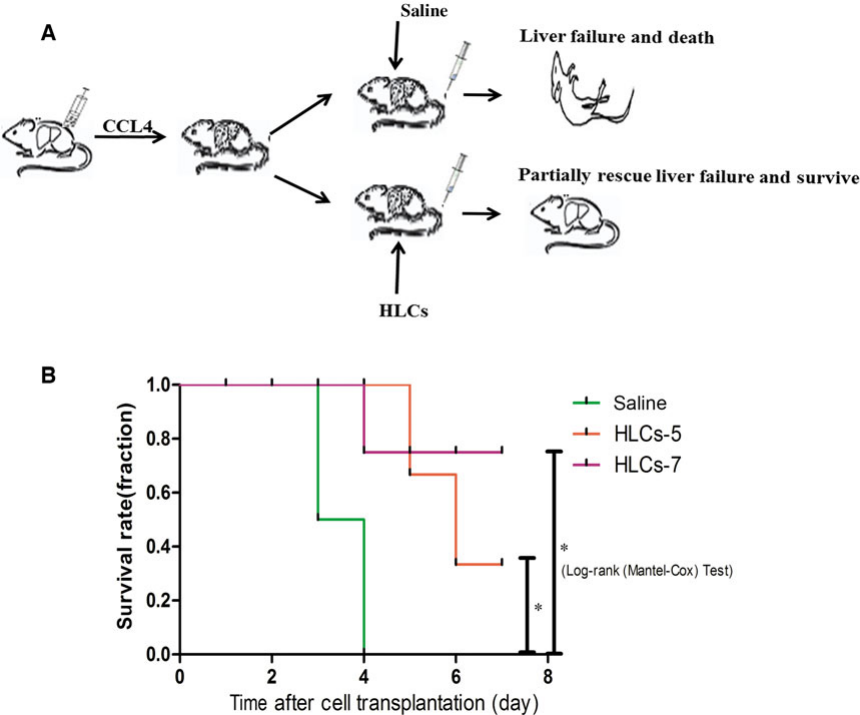
HLC‐5 transplantation extended the survival time of CCl_4_‐induced liver failure nude mice (*n* = 6). (**A**) The outline of the induced cells transplantation into the male nude mice with ALF. (**B**) Kaplan–Meier survival curve of CCl_4_‐injured mice that received or did not receive 1 × 10^6^ HLCs.

Next, we induced the ALF model with severe liver damage to give needed much attention on how HLC‐5 transplantation affected the survival condition. We examined the survival rates of this liver failure model within 1 week (Fig. 6A). One day after CCl_4_ treatment, the liver of mice exhibited a smaller than normal volume. H&E staining showed great damage of liver architecture and inflammatory infiltrations (Fig. S3B). All of the severe liver‐damaged mice treated with saline without HLCs died from liver failure within 4 days. Two of the mice in the HLC‐5 transplanted group died at day 4 and day 5. There was one mice of the HLC‐7 treatment died during the observation period. Survival analysis showed that compared with the saline control group, the HLC‐5 and HLC‐7 as positive control‐treated groups showed longer survival time. Surprisingly, HLC‐5 transplantation significantly increased the survival period of the severe‐damage mice as HLC‐7 transplantation (Fig. 6B). Together, these results indicated that HLC‐5 performed the hepatic functions of *in vivo* and provided evidence that HLC‐5 could function as HLC‐7 both *in vitro* and *in vivo*.

## Discussion

As the application of primary hepatocytes is hindered by their lack of proliferation and loss of function 5, the differentiation of human MSCs into HLC is a promising strategy for generating an unlimited supply of hepatocytes for BALSS. To date, the widely used method to induce hepatic differentiation of stem cells is to apply cytokines and growth factors. This method relies heavily on diverse growth factors, for example HGF, activin and dexamethasone. The cost, complexity of the method and long‐time induction are roadblocks for the use of resultant functional HLCs in BALSS.

MicroRNAs have been indicated to play pivotal roles during liver development and the hepatic differentiation of stem cells 20, 27. Previously, we elucidated the microRNA expression profile during hepatic differentiation of hMSCs and provided the basis for clarifying the roles of microRNA in differentiation process 25. Later, our group found that overexpressing a group of seven microRNAs (miR‐122, miR‐148a, miR‐424, miR‐1290, miR‐542‐5p, miR‐1246 and miR‐30a) could induce hMSCs to convert into HLC in only 7 days. Moreover, these differentiated cells performed liver functions *in vitro* such as LDL uptake and glycogen storage and significantly improved the liver function of injury mouse models *in vivo* 26. In this study, by screening the effects of candidate microRNA of seven‐miRNA pool, we identified a mixture of five miRNAs (miR‐122, miR‐148a, miR‐424, miR‐542‐5p and miR‐1246) that can convert hMSCs into HLCs. The induced HLC‐5 not only expressed hepatic markers, but also demonstrated LDL intake and glycogen storage, therefore resembling HLC‐7. It has been stated that true hepatocyte should contain hepatic marker expression and crucial hepatocyte‐defining enzymatic properties 28. Thus, we measured the levels of the CYP enzyme CYP3A4 and CYP1A1, which were important for detoxification of xenobiotic, and found that they were increased in HLC‐5 (Fig. 3A and Fig. S2E). Moreover, the level of CYP1A1 activity induced by omeprazole was greatly increased in HLC‐5, but lower than that of primary hepatocytes (Fig. S2F).

Additionally, we transplanted these HLCs into CCl_4_‐injured mice and surprisingly observed the improvement of serum parameters and the amelioration of liver histology in the HLC‐5 treatment group. Interestingly, the HLC‐5 treatment group exhibited survival improvement that closely mimicked that of the HLC‐7 treatment group as determined by comparing them with the saline control group. We also traced the transplanted cells after 1 week, in order to ensure that these cells were located in the liver. We considered the moderate rate of detected HLCs was due to that the limited quantity of biopsied liver samples.

The optimal therapeutic protocol to obtain seed cells for bio‐artificial liver support system should be rapid and highly efficient, because patients with severe hepatic failure are in urge need of therapy. The growth factors‐based method took four or more weeks before harvesting mature HLC 12, 25. Surprisingly, our protocol dramatically shortened the time for hepatic conversion to only 1 week. Moreover, the obtained cell population showed liver function both *in vitro* and *in vivo*. In our study, miR‐30a and miR‐1290 were not necessary for the hepatic differentiation process. The HLC‐5 had the similar gene expression profile and hepatic liver function as HLC‐7 *in vitro*. More importantly, the results of obtained studying CCl_4_‐induced liver injury and fulminant hepatic failure mouse models demonstrated that the treatment with HLC‐5 and HLC‐7 improved liver function and histology, without significant difference between them. We believe the differentiated HLCs derived by simplified five‐miRNA combination would be developed into an alternative cell source for BLASS.

Nowadays, the molecular mechanism and regulatory signals underlying the hepatic differentiation from MSCs are uncertain. It has been reported that transcription factors 13, epigenetic modification and mesenchymal–epithelial transition 29, 30 are closely associated with hepatic differentiation from stem cells. Additionally, various cellular pathways, such as wnt/β‐catenin pathway, may control the trans‐differentiation of MSCs 31. HNF4A was found to be a key factor in determining the differentiation of HLC from hMSCs, activating the expression of hepatocyte‐specific genes and enhancing the hepatic differentiation status 32. The microRNAs that we have identified as critical in this study have also been reported to be associated with these potential processes. MiR‐122, the liver‐enriched microRNA, promotes hepatic differentiation of liver progenitor cells *in vitro* 33. It was also demonstrated that miR‐122 could function as an effector of liver‐enriched transcription factors (LETFs) and targeted CUTL1 to contribute to mouse liver development 34. These results provide a basis for us to explore the detailed mechanism through which the miRNA combination induces hepatic differentiation. Besides, miR‐148a had been shown to play a pivotal role in the liver by promoting the hepatospecific phenotype 35 and participate in mesenchymal–epithelial translation (MET) 36. During the differentiation from mouse foetal hepatoblasts into mature hepatocytes, miR‐148a was identified highly expressed in adult liver and was reported to direct targeting of DNA methyl‐transferase (DNMT) 1 to control hepatic differentiation 35. Ectopic overexpression of miR‐424‐5p was sufficient to block epithelial–mesenchymal transition process by targeting the potent β‐catenin inhibitor ICAT/CTNNBIP1 37. Additionally, miR‐424 was suggested to regulate myofibroblast differentiation during EMT process 38. miR‐542‐5p was reported to be related with regeneration neonatal mouse heart 39. Overexpression of miR‐1246 could induce cell proliferation, because it regulates the expression of cyclin G2 which is associated with growth inhibition 40. In this study, we applied bioinformatics techniques and built up the network of mechanisms by which five miRNAs induce the hepatic differentiation of MSCs for further research (Fig. S4). So far, there are no reports illustrating the definitive mechanism through which miRNAs affect stem cell differentiation. Thus, additional research aimed at the application of HLCs derived from miRNAs is required.

In summary, our data demonstrated that hMSCs can rapidly and efficiently be converted into functional HLC by the transfection of five‐miRNA combination. With the regulation of miRNAs, we harvested cell population that not only produced urea, stored glycogen and took up LDL, but also improved the status of liver injury mouse models. This study provides experimental evidence for the application of stem cell‐derived HLC transplantation. Using our protocol, a reliable cell source for BALSS to clinical application for patients with severe liver failure might be obtained. However, further research is necessary due to the limitations of our study.

## Conclusion

We have established a simplified and optimized method to induce the hepatic differentiation from MSCs by a combination of miRNAs and found that miR‐30a and miR‐1290 were indispensable for this process. With the optimized miRNA group, we produced functional HLC‐5 cells, which will be a promising cell type for the application of BLASS.

## Conflict of interest

The authors declare no conflict of interest.

## Supporting information


**Figure S1** Identification and characterization of hMSC. (**A**) Surface markers of MSCs were identified by flow cytometry: CD31, CD34 and CD105. (**B**) Adipogenic differentiation of MSCs evaluated by oil red O staining. (**C**) Osteogenic differentiation of MSCs evaluated by alizarin red staining.

**Figure S2** (**A**) Hepatic differentiation efficiency of hMSCs mediated by different miRNA combinations. The percentages of LDL uptake‐positive cells were calculated after counting 200 cells. The percentages of glycogen storage‐positive cell were calculated after examining 200 cells. The percentages of ICG uptake‐positive cells were calculated after counting 200 cells. (**B**) Quantitative image analysis of Figure 3F. The results are expressed relative to a value of one in the control β‐actin. (**C–D**) Liver marker genes expressions of HLC‐5 and primary hepatocytes were analysed by qPCR. (**E**) The level of CYP1A1 was determined by qPCR. (**F**) CYP1A1 activity (EROD) of HLCs‐5 and primary hepatocyte.

**Figure S3** (**A**) The levels of serum parameters TBIL, IBIL and DBIL of liver‐injured mice after cell transplantation. (**B**) H&E staining of liver tissue from a) normal mice and b) CCl_4_‐injured exposed to 20% CCl_4_.

**Figure S4** Schematic showing the potential network of mechanisms by which five miRNAs induce hepatic differentiation of MSCs. Red square represents miRNAs. Blue circles represent mRNAs. Pink lines represent the targeted relationship of miRNA and mRNA. Green lines represent the protein interactions between different mRNAs.
