# Supplementary figures and images for: Induction of hepatocyte‐like cells from human umbilical cord‐derived mesenchymal stem cells by defined microRNAs
Source: J Cell Mol Med. 2016 Nov 22;21(5):881–93. doi: 10.1111/jcmm.13027 (PMC5387126; doi:10.1111/jcmm.13027)

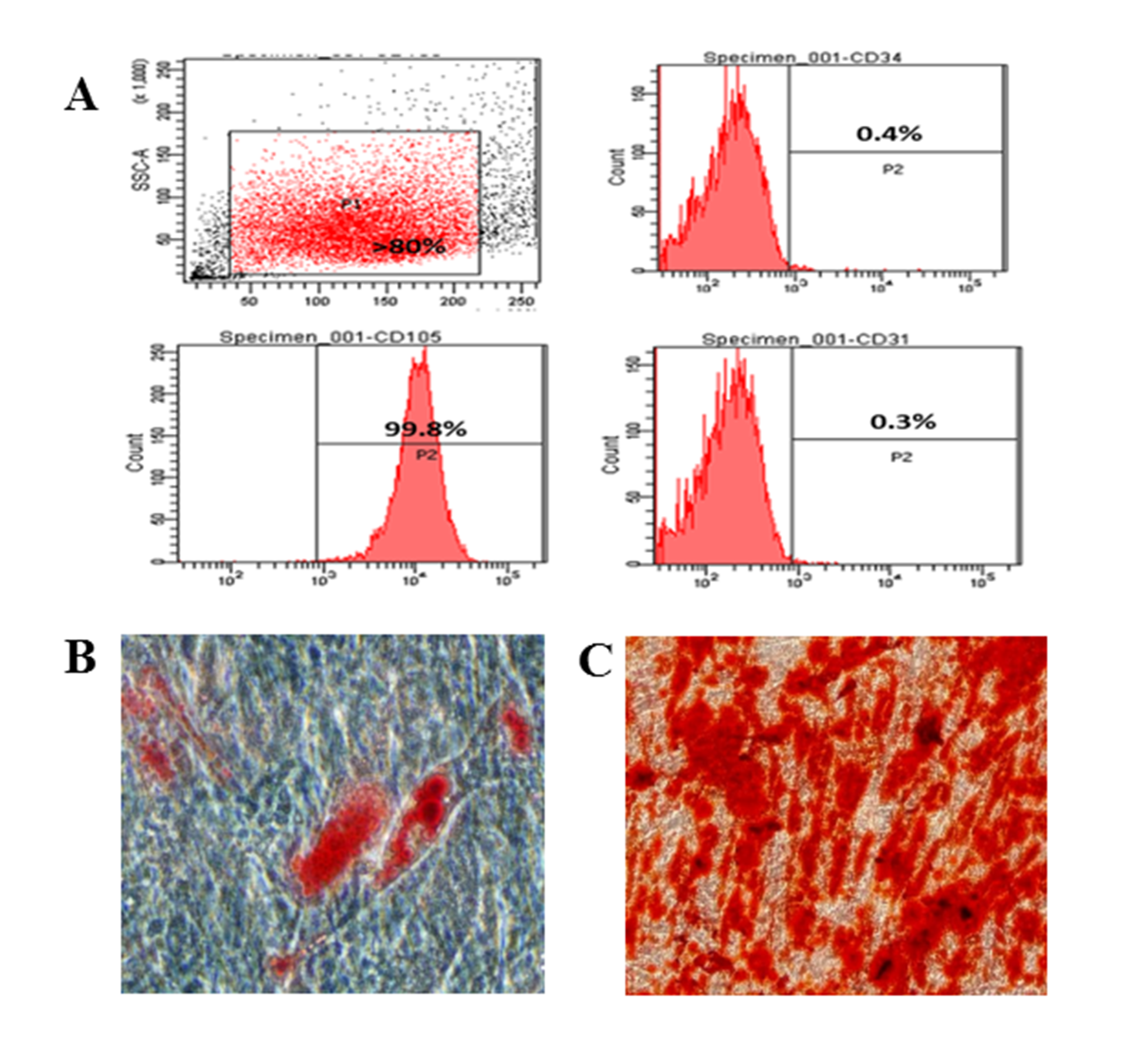

Supplement: Supplementary file 1 — Figure S1 Identification and characterization of hMSC. (A) Surface markers of MSCs were identified by flow cytometry: CD31, CD34 and CD105. (B) Adipogenic differentiation of MSCs evaluated by oil red O staining. (C) Osteogenic differentiation of MSCs evaluated by alizarin red staining. [file JCMM-21-881-s001.tif]

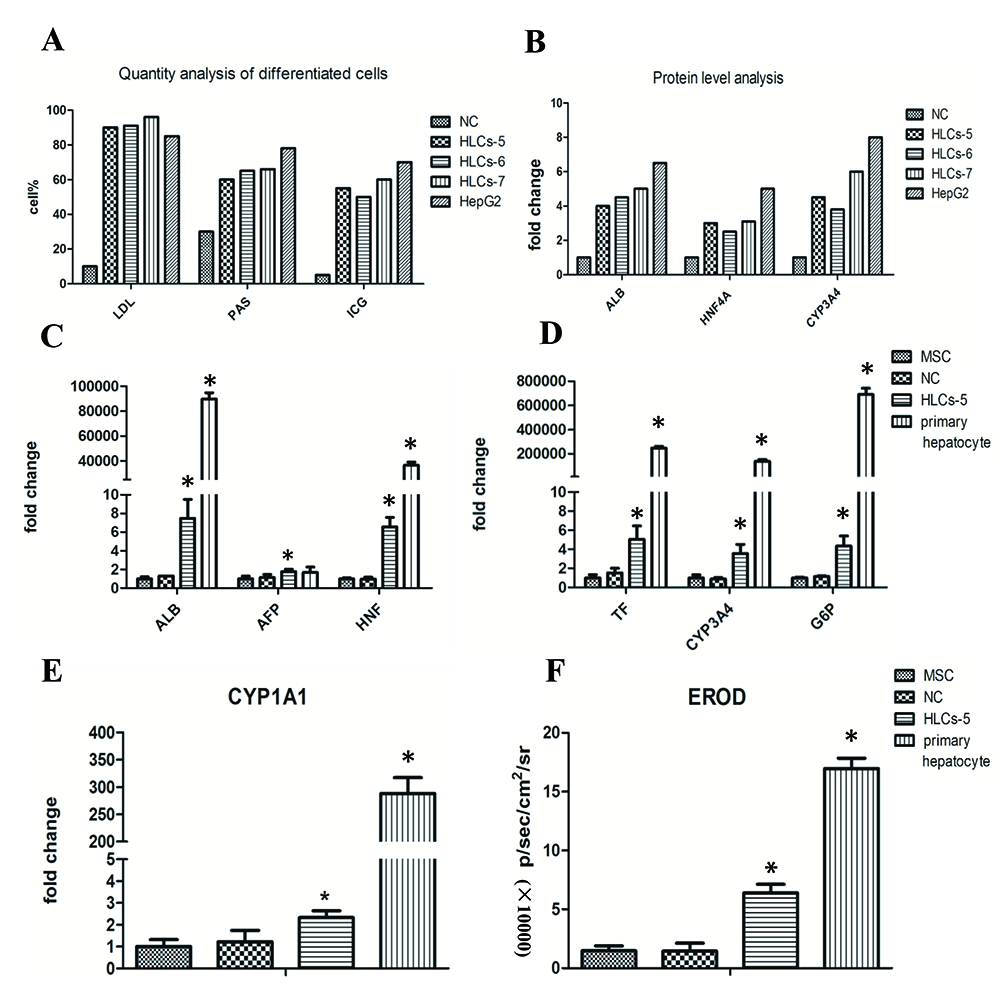

Supplement: Supplementary file 2 — Figure S2 (A) Hepatic differentiation efficiency of hMSCs mediated by different miRNA combinations. The percentages of LDL uptake‐positive cells were calculated after counting 200 cells. The percentages of glycogen storage‐positive cell were calculated after examining 200 cells. The percentages of ICG uptake‐positive cells were calculated after counting 200 cells. (B) Quantitative image analysis of Figure 3F. The results are expressed relative to a value of one in the control β‐actin. (C–D) Liver marker genes expressions of HLC‐5 and primary hepatocytes were analysed by qPCR. (E) The level of CYP1A1 was determined by qPCR. (F) CYP1A1 activity (EROD) of HLCs‐5 and primary hepatocyte. [file JCMM-21-881-s002.tif]

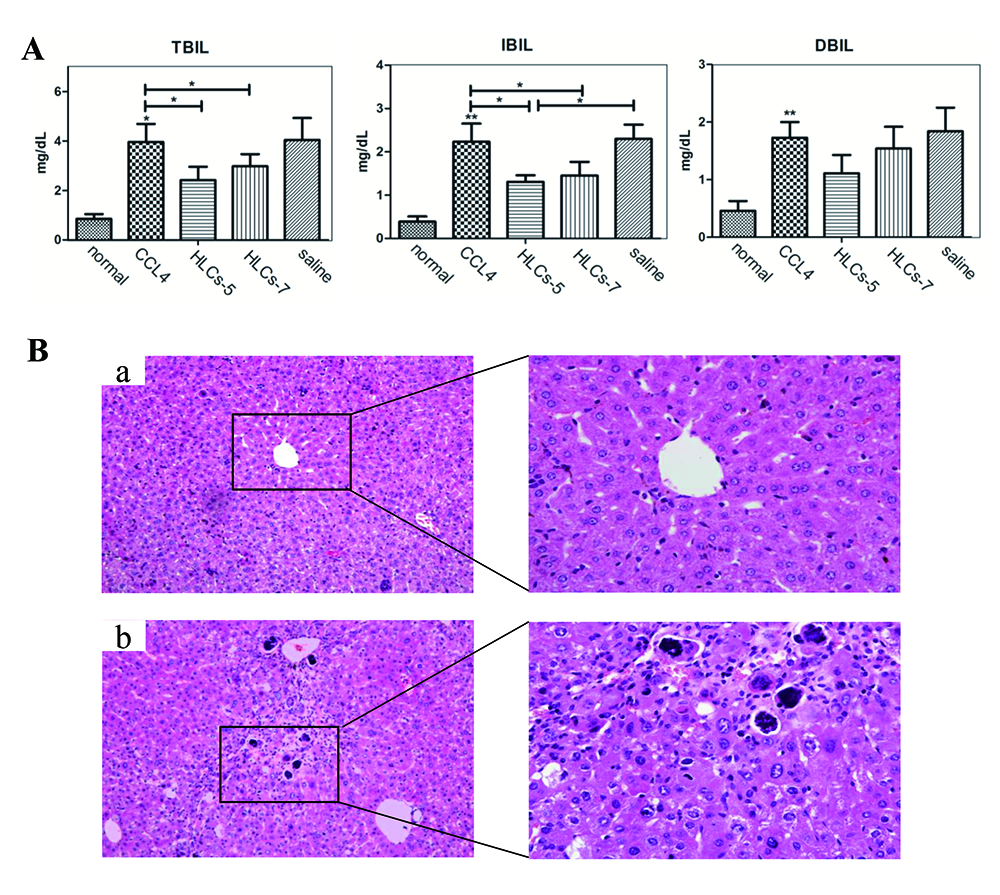

Supplement: Supplementary file 3 — Figure S3 (A) The levels of serum parameters TBIL, IBIL and DBIL of liver‐injured mice after cell transplantation. (B) H&E staining of liver tissue from a) normal mice and b) CCl4‐injured exposed to 20% CCl4. [file JCMM-21-881-s003.tif]

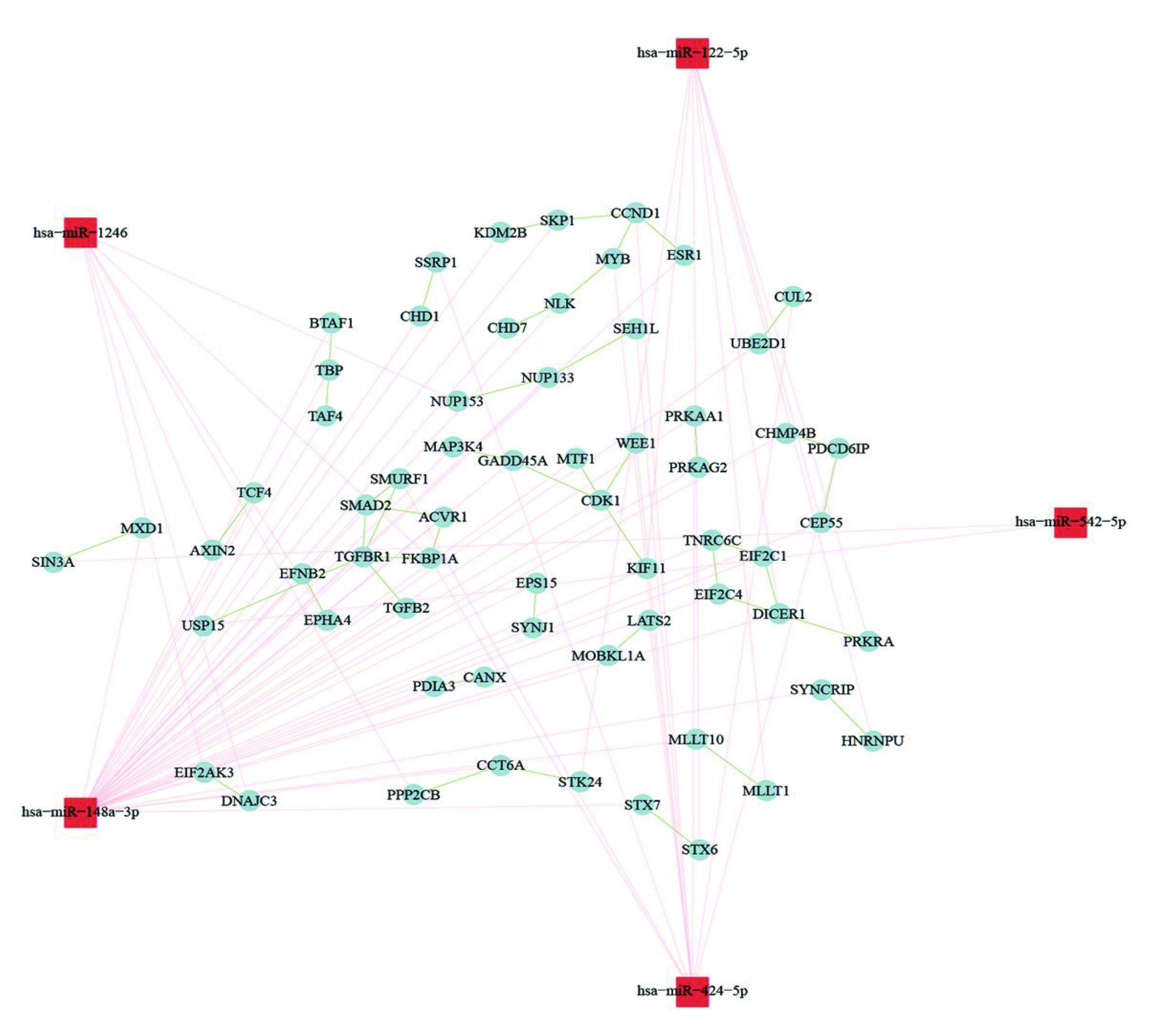

Supplement: Supplementary file 4 — Figure S4 Schematic showing the potential network of mechanisms by which five miRNAs induce hepatic differentiation of MSCs. Red square represents miRNAs. Blue circles represent mRNAs. Pink lines represent the targeted relationship of miRNA and mRNA. Green lines represent the protein interactions between different mRNAs. [file JCMM-21-881-s004.tif]
